# The Morphia Habit, and Its Voluntary Renunciation

**Published:** 1910-03

**Authors:** 


**The Morphia Habit, and its Voluntary Renunciation.** By OSCAR JENNINGS, M.D. Pp. x, 492. London: Baillière, Tindall & Cox. 1909.

This personal relation of a suppression after twenty-five years' addiction cannot fail to be of much interest to all medical men. The author in his preface states "that one medical man out of four is a drug *habitué*, usually a morphinist, that the proportion of medical addicts to the total of cases is in some statistics as high as 90 per cent., and that one-fifth of the mortality in the profession is said to be caused by morphinism." We cannot but think that these statements are grounded on fallacies of observation or statistics, as certainly they are not in harmony with general experience. Medical men are as a rule cautious in their use of active drugs, and discontinue them when the indications for them have ceased; satire usually credits them with a tendency to give drugs to other people but to take none of them themselves. However, whether the patient be medical or otherwise, he may learn from this book that "if he intends and wills to do so, he can cure himself."

